# Green Mind Theory: How Brain-Body-Behaviour Links into Natural and Social Environments for Healthy Habits

**DOI:** 10.3390/ijerph14070706

**Published:** 2017-06-30

**Authors:** Jules Pretty, Mike Rogerson, Jo Barton

**Affiliations:** 1 School of Biological Sciences, University of Essex, Colchester CO4 3SQ, UK; 2 School of Sport, Rehabilitation and Exercise Sciences, University of Essex, Colchester CO4 3SQ, UK; mike.rogerson@essex.ac.uk (M.B.); jobarton@essex.ac.uk (J.B.)

**Keywords:** green minds, green exercise, nature and health, healthy behaviours

## Abstract

We propose a Green Mind Theory (GMT) to link the human mind with the brain and body, and connect the body into natural and social environments. The processes are reciprocal: environments shape bodies, brains, and minds; minds change body behaviours that shape the external environment. GMT offers routes to improved individual well-being whilst building towards greener economies. It builds upon research on green exercise and nature-based therapies, and draws on understanding derived from neuroscience and brain plasticity, spiritual and wisdom traditions, the lifeways of original cultures, and material consumption behaviours. We set out a simple metaphor for brain function: a bottom brain stem that is fast-acting, involuntary, impulsive, and the driver of fight and flight behaviours; a top brain cortex that is slower, voluntary, the centre for learning, and the driver of rest and digest. The bottom brain reacts before thought and directs the sympathetic nervous system. The top brain is calming, directing the parasympathetic nervous system. Here, we call the top brain blue and the bottom brain red; too much red brain is bad for health. In modern high-consumption economies, life has often come to be lived on red alert. An over-active red mode impacts the gastrointestinal, immune, cardiovascular, and endocrine systems. We develop our knowledge of nature-based interventions, and suggest a framework for the blue brain-red brain-green mind. We show how activities involving immersive-attention quieten internal chatter, how habits affect behaviours across the lifecourse, how long habits take to be formed and hard-wired into daily practice, the role of place making, and finally how green minds could foster prosocial and greener economies. We conclude with observations on twelve research priorities and health interventions, and ten calls to action.

## 1. Recent Findings from Research on Nature and Health

We have undertaken over a decade of research into the contexts, effects, and outcomes of *green exercise* and nature-based interventions, showing in a wide variety of contexts that physical activity in the presence of nature improves health and well-being [1,2,3,4,5,6,7,8,9,10,11,12,13,14], as have other authors [15].

We have found no groups who have not benefitted: all ages, genders, ethnicities, and social classes respond positively to green exercise. We have shown that all natural environments are beneficial: from urban parks to biodiversity-rich ones, from small local to large landscapes, and from domesticated gardens to the farmed and wild. We coined the phrase *dose of nature* to articulate that exposure to green exercise is analogous to a medical dose to the body, improving mental health [16] (see also [17]). We have shown that the deliberate therapeutic use of natural environments (e.g., gardens, allotments, care farms, and wild places) has both short and long term positive effects on groups under mental stress, including at-risk children and youth, refugees, probationers, dementia sufferers, office workers, and mental health patients. The natural environment is now understood to provide vital health services as well as other environmental services [12,18,19,20]. These health services include direct and indirect effects on physical and mental health, and reductions in the threats of pollution and disease vectors [18].

Further research has filled many gaps, exploring inner mechanisms, external social processes, interactions with place and behaviours across the lifecourse. It has been shown that greener environments are equigenic, reducing social inequality and having particularly positive impacts on mental well-being [21,22,23], that physical labour in the home is important for health and longevity [24,25], and that blue space (locations in sight of water) is as important as green: it is not the colour that matters, but the opportunity to behave in a way that improves well-being [26]. A meta-analysis of nature connectivity and well-being [27] has shown the more connected to nature a person is, the greater is their life satisfaction.

At the same time, the design of human settlements and buildings influences human health [28,29,30,31], suggesting that natural places can be thought of as healing places [32,33,34]. Typical urban settings are more discomforting, with metabolic and well-being consequences [35,36]. Exposure to nature reduces internal stress markers and produces healthier cortisol profiles [37,38]. Knowing is important too. knowledge of being treated (both dose of nature and drug medication) causes a release of endogenous opioids that are non-addictive: the placebo effect demonstrates the mechanisms for self-healing (of some conditions) [39,40].

Lifecourse and longitudinal studies (e.g., Caerphilly men, Dunedin, Maudsley and Cambridge cohorts, Milwaukee nuns, Harvard alumni) have shown how choices on behaviours, consumption, and mental states directly affect health and well-being over many decades [41,42,43,44,45]. These studies demonstrate the value of early interventions with children whose cognitive outcomes are improved when regularly exposed to activity in natural places (playgrounds, gardens, and woodlands) [46,47].

We have calculated the annual health costs of seven lifestyle-related conditions in the U.K. (obesity, type 2 diabetes, loneliness, cardiovascular diseases, mental ill-health, dementias, and physical inactivity), all of which are influenced by a lack of physical activity, links to natural places, and links to community and people; annually these amount to around £180 billion. All of these conditions are influenced by a lack of physical activity, links to natural places, and links to community and people, with annual health costs ranging from £8–105 billion (Table 1). This suggests that high levels of material consumption in affluent countries have not necessarily brought increased health and well-being for all; that consumption patterns in most countries of the world are converging on those typical to the affluent; and that new ways of living are required that emphasise non-material consumption if planetary and personal harm is to be avoided [5,13,48].

Many of the drivers of ill-health in Table 1 are behaviour- and lifestyle-related: too many calories consumed in food and drink, too little physical activity, and too little social engagement. Through a variety of interventions, there is now the prospect of the U.K. becoming virtually tobacco-free (<16% of the adult population now smoke tobacco, down from 50% in the mid-1970s). Yet the implementation of healthy food, activity, and engagement activities for whole populations seems impossible, despite advances on nudge tactics (a concept that involves positive reinforcement to achieve non-forced compliance) [50]. It is clear that social and economic environments do shape behaviours. Residents of London walk 292 miles per year; but rural people walk just 122 miles. Obesity afflicts 35% of adults in the U.S.; in Manhattan, where there are pavements and public transport, people walk more, and only 15% are obese. In the Japanese, Sardinian, and Costa Rican longevity hotspots, cultures encourage healthy and tasty foods, regular physical activity outdoors, social connections, and continued cognitive engagement [24,51,52]. Individual choices do not arise from failures of free-will, but are shaped by the interactions between the design of lived environments, transport systems, institutional inertia, advertising and corporate self-interest, and access to green space. Here, we explore how behaviours benefitting health can be adopted and encouraged.

## 2. Red Brain, Blue Brain, Green Mind

We are proposing a Green Mind Theory (GMT) that links the human mind with the brain and body, and connects the body with natural and social environments. The processes are reciprocal: environments shape bodies, brains, and minds; minds change body behaviours that shape social interactions and natural capital. GMT offers opportunities for improving individual well-being whilst building towards greener and prosocial economies that could protect the planetary future. The empirical understanding of green minds derives from evidence on neuroscience and brain plasticity [53,54,55,56,57,58], from spiritual and wisdom traditions [59,60,61,62], from mindfulness-related and talking therapies [63,64], from green exercise activities in nature [1,13,16], from the lifeways of indigenous groups [6,65,66,67], and from material consumption behaviours and the potential emergence of green and prosocial economies [5,14,68,69].

We outline how and why pathways of poor health can be engaged by surrounding natural and social environments in ways that promote reciprocal individual-society-natural well-being. Our concern is not to assume a comprehensive description of many complex brain-body interactions, nor to take a simple behaviourist model whereby well-being or happiness can appear to be guaranteed. Our desire is to use an emerging understanding of brain-body-behaviours to develop pathways and interventions for better health and well-being.

We propose a simple brain metaphor with levels representing different stages of mammal-hominid evolution [54,56,57,58,70]. The brain stem (bottom brain) is the oldest and contains survival functions: it is fast responding, involuntary, automatic, impulsive, driven by emotions, and is the executor for habits and routines. In the mid-brain, the limbic/sub-cortex system is the central region comprising the hippocampus, hypothalamus and thalamus, and amygdala: it is the centre for emotions, memory forming, and bonding. The top-brain cortex is the most recent, having expanded in size rapidly during the later stages of hominid evolution: it is slower, voluntary, able to learn and plan, make internal choices, and contains centres for the social abilities of empathy and language.

The top brain is calming, drives the parasympathetic nervous system (PNS), and is characterised by rest-and-digest. The bottom brain is spiked into action by the amygdala, the sentinel for emotional meaning, which drives the sympathetic nervous system (SNS) via the hypothalamus pituitary adrenal axis (HPAA), and is characterised by fight-and-flight. We call the top brain blue, and the bottom brain red.

We use the term *green mind* to suggest an optimal mixed mode of mainly activated PNS, interest and excitement-associated mild SNS stimulation, and the presence of only occasional SNS spikes for alarm response. A mix of blue and red is best for health and well-being. Too much red brain is detrimental for health. The key endogenous neurotransmitters, hormones, and peptide pathways are serotonin (key to sleep and mood); dopamine (aids approach, attention, and rewards, but when it falls it makes us feel unpleasant); norepinephrine (promotes alertness and arousal); acetylcholine (promotes wakefulness and learning); opioids (reduces pain and buffer stress); oxytocin (a key role in bonding and feelings of bliss); cortisol (stimulates amygdala and inhibits hippocampus); oestrogen (key in memory); and adrenalin (stress hormone). Our proposal for the greener mind centres on activities that bring immersion-attentiveness, so calming the mind. We will later show how these centre on the choice architecture for engagements with nature, with other people, and with craft-skill based activities. Green Mind Theory suggests that desirable fundamentals to well-being are in reach of everyone. Yet the negativity bias of the brain has become dominant in modern living. A summary of the key differences between the modern and green mind is shown in Table 2.

## 3. The Brain’s Negativity Bias and Causes of Suffering

It is plausible that natural selection has built a negativity bias into the brain-mind [57]. The amygdala responds immediately to alerts, and succeeds by being over-responsive. In evolutionary history, to miss one threat meant death; to miss one positive signal was not necessarily critical. The brain-mind thus evolved fast-acting and over-responsive fight-flight as the default mode. There is no moderation to the amygdala. It is a binary responder: fully on or off, and responding before thought. In hunter-gatherer-cultivator communities (the old economies: [71]), sites for millions of years of prior evolution, the blue brain mode tends to dominate [6]. The predator or poisonous snake was rare; threats from other hominid bands also scarce. By contrast, in modern affluent cultures dominated by material consumption and dashed hope, the red alert mode is regularly activated. Modern life appears to be lived on simmer, producing almost non-stop SNS-HPAA activation.

Repeated SNS-HPAA stimulation leads to an over-reactive amygdala, resulting in high anxiety and the continual shading of past memories with fear and anxiety. The current world gradually looks and feels worse, as does the past. The hippocampus is worn down, and memories harder to retain. Cortisol further suppresses new neurons in the hippocampus. Too much SNS-HPAA has negative impacts on the gastrointestinal system (more ulcers, inflammatory bowel syndrome), on the immune system (more colds and flu, slower wound healing), on the cardiovascular system (hardened arteries), and on the endocrine system (producing type 2 diabetes) [72,73]. Stress manifested as conditions (e.g., post-traumatic stress disorder (PTSD), depression) and illness (e.g., cardio-vascular disease) manifests internally as accumulation over long time periods of gradually worsening internal responses to the same stressors. Over time, continuing red brain activity exacts a cost, accelerating disease mechanisms, especially CVD, and the atrophy of brain structures (especially the hippocampus). Repeated hits of stress can leave stress hormone levels continually high, with no recovery period.

Modern living is often characterised by cognitive overload, impulsive habits, and individual behaviours that have led to a new generation of health challenges [13]. Just as shortages of food—eras of never-quite-enough—were solved in the 1950s–1970s in affluent countries, so food over-consumption became a leading problem [74,75]; just as eras of challenging transport requiring high energy expenditure ended, so inactivity and sedentary behaviours became common health problems. With these have come increased anxiety, guilt, and stress [13,56]. In a cosy modern world, many have forgotten discomfort [76], finding it easy to habituate over-eating or drinking, or a reliance on pharmaceutical interventions. In time, the habits evolve into pre-emptive strikes: promoting consumption before the anticipated discomfort.

Some spiritual and wisdom traditions call the red alerts *first arrows* [60]: these are fired by the amygdala and cannot be avoided. The *second arrows,* comprising how we feel in response to the first, commonly result in feelings of unfairness, guilt, further fear, anger, upset, and anxiety. Sometimes second arrows arise from anxious expectation, even when there has been no first arrow [56,57,77]. Threat signals are effective precisely because they are unpleasant. They should be: they make us suffer.

The first arrow threat alarm causes the amygdala to stimulate the thalamus, which sends norepinephrine to the brain stem (red brain). The SNS signals organs and muscles to ready for fight-flight, the hypothalamus now signalling the pituitary to instruct the adrenal glands to release adrenaline, cortisol, and epinephrine. The body is now on red alert, the epinephrine having increased heart rate and dilated pupils for more light, the cortisol suppressing the immune system and hippocampus. The executive control of the prefrontal cortex is switched off. Suffering arising from first arrows is thus embodied, and works through the cascade of SNS and HPAA. Most of the time, we hardly notice this is happening.

Modern affluent countries contain more lonely people, and have a growing number of adults living alone [13,78]. Lonely adults display elevated cortisol and epinephrine levels, and have higher blood pressure; the more active SNS-HPAA results in poorer sleep and lower immune function; it accelerates physiological decline with age [79,80]. Further linking the brain with behaviour, individuals with smaller social networks tend to have a smaller amygdala and hippocampus: structures that play an important role in social behaviour [81]. The lonely are red brain dominant, and their lifespan is reduced. In modern living, first arrows have become more common, and together with second arrows have negative impacts on memory, general well-being, and habit forming. A strong blue brain is able to dampen second arrows.

## 4. Immersion and Attentiveness to Quiet the Chatter

A green mind should have a healthy mix of brain states, with a predominance of calm blue. A range of different descriptors have been used to describe a state of mind that results in a temporary dampening of second arrows by building up the PNS: *focus*, *attention*, *awareness*, and *immersion*. There is clear evidence to show that activities that are immersive and involve focused attention are effective in improving well-being [54,68,82]: they cause instant physiological changes by reducing oxygen consumption, lowering heart rate and blood pressure, and increasing the release of serotonin and dopamine [72].

The importance of recovering the capacity to focus attention was first proposed by Kaplan and Kaplan [83], leading to a growing understanding of the restorative benefits of nature [84,85]. A variety of stressors cause attention fatigue: Attention Restoration Theory showed how focused attention, with positive well-being outcomes, could be recovered or restored by engagement with a variety of environments, both natural and non-natural [86,87]. More recently, research on salutogenesis, the range of factors supporting health and well-being, has developed an understanding of how environments can both deplete and restore internal resources [29]. Here, we suggest it is the immersion and activation of the PNS that is the key mechanism. Some environments are thus seen as restorative, as are some behaviours and activities.

We know that immersive-attention is an element of some green exercise, arising during walking, moderate running, and gardening [4,88]; during meditative activities such as yoga, tai chi, and mindfulness [64,77,89,90,91,92]; and during many craft and skill-based activities such as woodwork, painting, knitting, and needlework [68,93]. In some, exercise involves no more than sitting; in others, it is part of cultural events: community dancing and singing of *sennin* of Tibet, the *haka* of Māori in New Zealand, the chain dance of Faroe, the *reimur* of Iceland, the shadow puppetry of Indonesia, the ceremonial dances of American Indian tribes, and forest bathing in Japan [94].

Being highly attentive could have brought evolutionary advantage to hunter-gatherer-cultivators. Watchful awareness is central to the hunt; is vital for caring for plants and animals across the seasons; is critical to memory creation for sources of water and signals for weather events. Hunter-gatherer-cultivators spent large amounts of time waiting, observing keenly, and preparing and eating food. Yet in material cultures and economies where a life on automatic seems a modern malaise, millions actively choose opportunities for quiet and calm: watching sunsets, beach holidays, being with friends, and activities that require focus and take time to learn.

A second feature of a brain dominated by second arrows is the endless chatter that originates in the verbal and language centres of the left prefrontal cortex. This chatter comprises the loops of internal voice that act as commentary on past events, future possibilities, and current concerns [56]. The autobiographical self runs loops of past and future, and is often self-critical. Anxiety retones memories of the past, gradually making them feel worse. Immersive-attentive activities are calming and take time. They activate the PNS, and increase neurone growth in the insula, hippocampus, and prefrontal cortex, particularly the left prefrontal cortex (PFC) where feelings of well-being reside. A steady release of dopamine is produced, increasing a sense of well-being. The PNS further decreases cortisol and strengthens the activity of the immune system. Immersive-attentiveness can thus feel like a high vantage point, being on a hill looking down on the distant valley below, or on cliffs looking at the silent sea. When the green mind is quiet, the self is stilled. A wandering mind is an unhappy mind [95].

## 5. Habits and Behaviours in the Lifecourse

Planning and learning occurs in the PFC, and as routines are automated so they are sent downwards to the mid and lower brains, and tend to remain fixed unless brought back up for specific improvements. As we habituate a routine more, so the basal ganglia take over from the PFC and automate the routine, allowing us to pay less attention (and use up less energy). This occurs in the learning of language, the gait of walking, the poise of sitting, preferences for foods, and the mode of driving a car or riding a bicycle. All require hours of practice, but once learned no longer require active attention. Many of the modern conditions of ill-health result from behaviours gradually adopted over time and are thus hard to challenge, including eating and drinking habits, smoking, sedentary lifestyles, reduced direct contacts with family and community, less active transport, reduced contact with green places, and increased use of pharmaceutical solutions to ill-health [13].

Will-power and focus are vital capabilities for learning skill-based habits. To learn a language (the first or more), arithmetic and times tables, to drive a vehicle, all require practice. The greater the cognitive control by the PFC, the less anxiety we suffer. In the Dunedin longitudinal study, children with the greatest will-power early in life had the best health outcomes during the lifecourse. The critical years for children to learn self-control are 5–8 years of age: if they do, this improves cognitive capability [96]. Time outdoors as a child facilitates, and also predicts, adult health [97].

One enduring heuristic suggests it takes ten thousand hours to learn to become an expert [98,99]. Yet for most behaviours it is far fewer, as repetition sooner sends routines to the bottom brain. Repeated behaviours in consistent settings proceed more efficiently, and are strengthened through association between situation (environmental cues) and action [100,101]. Experimental research has shown it takes 28 to 84 days to form a habit, depending on time spent per day, or ten weeks for healthy habits to become fixed [100,101,102]. Thirty minutes a day for 30 days of time in nature has been shown to improve well-being, mood, and mindfulness, though for this to become fixed as a habit, it is likely to need three times the number of days for long-term adherence [17]. Weekly interventions of walking and tai chi for 40 and 52 weeks have been shown to increase hippocampus volume in the elderly [103,104].

We thus suggest a rule of thumb of 50 days at one hour per day, or 100 days (approximately 3 months) at half an hour per day, to produce changes in the brain and result in fixes to behaviour. To improve or change a habituated routine, it needs to be brought back to the PFC, evaluated, and amended. But changing ingrained habits is hard: we have to force the body to do something different as it will not choose voluntarily to do so. Habits must be brought back up to the blue brain, and become subject to specific attention.

In the increasingly typical modern lifecourse, many people also give up activities that brought them pleasure on the grounds that they have neither the time nor the energy to continue. Over the lifecourse, inch by inch, we cede territory to automated behaviours and habits that often bring discontent. We pay less attention to eating well, forget friends, and become less active. Halpern [50] posed a question: when did you stop dancing? One significant policy and practice challenge is to identify the habit-releasers for improved health: new behaviours need forceful and sometimes fierce action. Another centres on the choice architecture of social and cultural environments that in turn shape choices about behaviours [50]. The U.K.’s Behavioural Insight Team, or Nudge Unit, has demonstrated how small nudges can lead to shifts in behaviours across large populations. But frequent practice is still required to fix new patterns in the brain: the fifty hours. The key concept is that the brain changes as a result of body behaviours: the property of neuroplasticity [58].

## 6. Neuroplasticity and Placebos

Neuroplasticity is the property of the brain to change structure and function by responding to actions by the body, to signals received from the external world, and to mental experiences. In this way, external and internal signals are not materially different: they are just signals. It is also understood that neurons that *fire together, wire together*; and those not used will die back. The conventional predominant view of the brain-mind is that if it breaks down, nothing can be done. The concept of neuroplasticity suggests new opportunities for directed or chosen changes across the lifecourse.

The first example centres on pain and pain control. Acute pain is a signal to attend to a problem immediately. But neuropathic-chronic pain is different: it comprises the incessant false alarms of the after-life of acute pain. These repeated mental experiences cause structural changes in the brain, and the pain map continues expanding, and invading parts of the brain that process thought, sensations, images, and memory [57,58]. Persistent and chronic pain is also demoralising [105], setting off new red alerts from the amygdala. An understanding of neuroplasticity has shown that the pain gate can be raised by endorphins, and these can be released by immersion-attentiveness activities such as mindfulness, meditation, and tai chi [106]. The gate rises, the pain sensations fall, and counter-stimulations in the invaded parts of the brain push away the pain memories. However, individuals have to be relentless in forming this new habit: they have to be more relentless than the pain that produced the map [58].

The placebo effect (PE) is a second example, and is no less real because it is driven by thought. It causes as many changes in the brain as does medication. The placebo has long been conceptualised as an inert process, and thus used as an experimental control for drug testing. But recent research on the PE has shown the potential benefits of self-healing. The PE is a genuine phenomenon driven by expectancy in both patient and physicians/nurses, and has yielded beneficial clinical results for angina, bronchial asthma, herpes, ulcers, inflammatory bowel syndrome, and persistent pain [39,40,107]. The PE mechanism centres on the self-release of non-addictive endogenous opioids.

It has been noted that alternative therapies with no clear scientific explanation but with elaborate rituals and beliefs can thus induce placebo effects, particularly if there is a good personal relationship between the practitioner and the patient [40]. It has also been shown that treatment augmented with warmth, attention, and confidence improves clinical outcomes. Patients thus engage in treating themselves if physicians, nurses, and carers have a friendly manner, engage in active listening, show empathy, allow periods of silence in conversation, and communicate confidence and positive expectations [108]. The British Medical Association [109] has reinforced the importance of compassion and empathy for patient-centred care. In noting the high levels of boredom on hospital wards, where there is too little physical activity, they have recommended hospitals engage in deliberate social activities, such as creative writing, music, visual art, dance, and singing. Better design would help too, including for creating healing gardens [33].

## 7. Place Making

The green mind suggests that individuals link to natural places that are recognisable and individualised. These might include urban parks, gardens, nature reserves, and walking routes (including for dog walkers). In the contemporary affluent world, chronic placelessness has become endemic. People spend less time outdoors, travel less by walking and cycling, and move house more often [5,14]. Children’s disconnection from natural places stores up future problems, as fewer memories are made of life events in the critical middle age of childhood from 5 to 11 years [110]. The structure of physical and natural environments is now well-established as having an impact on physical and mental health [20,22,23,34], with physical activity in cities by cycling and walking reducing cancer risk [111].

In an enriched environment, new neurons are produced by the hippocampus, which then turns short-term memories to long-term ones. Moderate activity, for example walking and tai chi, also produces new neurons in the hippocampus [112] and increases hippocampus volume, thus improving memory [103,104]. Walking forwards into landscapes thus creates long-term memories; walking has also been shown to protect against the neurodegeneration that causes Parkinson’s Disease (PD) and Huntington’s Disease, delaying the onset of dementias by ten years. A sedentary, immobile lifestyle is less stimulating, causing parts of the brain to atrophy. A central problem in PD is inactivity, and medical treatment encourages passivity. Walking can help to cure it, but may take a high degree of concentration to bring habits from the bottom brain to the top [113]. Exercise in natural places is as effective as fluoxetine (e.g., Prozac) for many people [114], and tranquil scenes can quieten the mind [115].

For better health and well-being, many people thus need behaviours that increase both memory- and place-making. Places are dense with meaning, stories, memories, and morals. They work on your mind [6,65]. The contours of our minds are shaped by particular places. Both gardening and allotmenteering promote recovery from stress and improve well-being; members of allotment groups experience less stress than same-age members of indoor groups [4,116].

Location can improve health, especially if the place is culturally considered as home [32]. Aboriginal groups returning to outstations have seen reductions in hypertension and diabetes [117]. Changes in well-being have been noted for Innu groups returning to the land in Labrador [118]. Aboriginal people describe land as a spiritual place, calm and centred, where the “quietness speaks to you” [119]. Natural and physically-sensate environments appear to give more opportunities for immersive-attention where there is a mix of mild continuing stimulation of the SNS and majority control by the calming PNS.

Simms and Potts [120] have argued for a *new materialism* in which is cultured a more pleasurable and respectful relationship with the world of things. Experiential purchases produce more enduring happiness than material purchase, with consumers deriving more benefits from anticipation, from the doing-experience, and from the memories created [121]. Finding fault with material consumption may appear to be seeking a return to living in a cave: it should not. The greater challenge is to increase individual well-being, reduce anxiety and depression, take more responsibility for the planet’s future, and create an abundance of less [122,123].

## 8. Linking Greener Minds to Contemplative and Greener Economies

Green Mind Theory offers an opportunity to link individual well-being to life behaviours and thus to whole economies. The future of the planet’s natural capital relies on new patterns of material consumption that shift behaviours to sustainable consumption (activities that build natural capital rather than deplete it), and/or non-material consumption (activities with a light footprint on resources, but which deliver well-being, such as listening to bird song, gardening, talking, walking, and volunteering) [5,124,125].

Green minds can build empathy and trust. They strengthen mirror neurons that show empathy, and oxytocin increases bonding between individuals and suppresses the red alert nature of the amygdala [126,127]. Some traditions call this increasing the *circle of us*, acknowledging the secret history of our enemies: they too feel sorrow and suffering, and ten thousand things may have caused them to act. A green and prosocial mind puts an emphasis on giving, contributing, and volunteering. Volunteers have higher well-being than non-volunteers, greater life satisfaction across the lifecourse, and live two years longer than non-volunteers [128,129,130].

Empathy would have been highly selected within group and bands during hominid evolution, and we search for it today. In hospital settings, patients are already on red-alert: they are anxious and worried. In the U.S., those surgeons sued the most do not make more mistakes, they were just unable to establish trust and empathy with patients [56]. Nurses and doctors who spend more time with patients smile and treat them as individuals, and are themselves happier; those patients also recover more rapidly and need less pain control. *Prosoche* is a component of many contemplative traditions: acute attention to and immersion in the present moment [61]. Prosoche moves in two directions: inwards into the mind, and outwards to the natural world and other people. More equal societies do better for all; inequality is bad for all [131,132]. Optimists live 19% longer than pessimists, suggesting that expectations about the future affect current well-being [133].

A popular assumption for the past half century has been that increased material consumption and rising GDP inevitably increases well-being. There have been many technological improvements to lives, yet timeless consumer culture invents new pleasures, often producing more suffering from second arrows: either we cannot access apparent pleasures, or when we do have them, they deliver less than expected, rapidly losing their lustre. One priority is to redefine prosperity, and by substituting activities that improve social cohesion, mental and physical well-being, and memory creation, the impact on natural capital and ecosystem services could be reduced whilst improving well-being [13,124].

Green growth and the green economy have become important targets for national and international organisations, including the OECD, UNEP, the World Bank, the Rio+20 conference, and the Global Green Growth Initiative [23,134,135,136]. UNEP [137] defines the green economy as “*resulting in human well-being and social equity, while significantly reducing environmental risks and ecological scarcities*”. To date, many countries acknowledge the need for greener economies, but few have acted significantly. Notable exceptions include: China’s launch of *eco-civilisation* policies, Korea’s building an advanced carbon economy, Kenya’s use of feed-in-tariffs dramatically to increase renewable sources of energy, and Denmark’s production of more energy from wind than it consumes nationally.

Greener economies will not look much like the current economy. They will be disruptive [138], but less than the impact of severe climate change. The notion of a greener and prosocial economy further implies a cultural understanding of how much is enough [139]. A key challenge is how a mode of consumption based on *enough not more* can be created, so resulting in mass behaviours of *enoughness* [135]. Wood et al. [140] have demonstrated the value of gratitude to well-being, and how it arises both from the receipt of aid/support from others and from an internal appreciation of the positive aspects of life. They conclude that people would be better off spending time amassing friendships and appreciating what they have rather than seeking higher incomes and amassing material possessions.

In greener economies, different forms of contemplative consumption will be valued, such as of story-telling, engaging with nature, and skill-based crafts. They tend to be cooperative, enhancing social capital formation and reducing inequity. This will bring positive feedbacks, as prosocial behaviours cause others to be prosocial, thus building social capital [141]. They offer four options to consumers: resist consumerism by opting out (e.g., downshifting, voluntary simplicity), retain possessions for longer (before replacement), make different choices (ethical or green consumerism), and substitute non-material consumption activities (e.g., nature consumption) [5]. Part of the solution for greener economies is the adoption of activities that lead to green minds. The Caerphilly Cohort Study has, though, shown that there was no change in the adoption of healthy behaviours by men over 30 years (commencing 1979): those with four of five behaviours (non-smoking, acceptable body mass, high fruit and vegetable consumption, regular activity, and low-moderate alcohol intake) delayed onset of heart disease by 12 years alongside reduced cognitive impairment and a delayed onset of dementia [42]. But those starting with low adoption did not change over time, even though public knowledge of these risks has grown. Kvaavik et al. [142] showed in a population of 5000 adults that the adoption of healthy behaviours extended life by a mean of 12 years.

## 9. Priorities for Implementing Healthy Habits

What does the Green Mind concept suggest for instruction and implementation? We have observed that the future of the planet relies on substitution of non-material consumption, making more of activities with a light footprint, and the co-delivery of well-being. But the history of implementation of healthy habits in affluent countries is not encouraging. There are five levels for possible action:

1. *International agreements*: these are rare, slow to implement, and easy to free-ride or undermine;
2. *National policies*: few successes for whole populations, so far, though anti-smoking and seat-belt legislation are successes;
3. *Institutional and sectoral policies and practice*: the potential for government, employers, and charitable organisations to change practices to affect large numbers of people, such as in education, mental health, social care, or hospitals;
4. *Community actions and ceremonies*: already widespread and manifested in local groups and rituals, but undervalued and not yet widely used to improve well-being;
5. *Voluntary actions of individuals*: hard to sustain, though the most commonly pushed by governments.

Elements 1 and 2 are hard to achieve, and 5 is often too easy to demand: it will be institutions and communities that are likely to reach the largest number of people quickly. We propose an emphasis on nature, social, and craft engagements in neighbourhoods, schools, care homes, and health care facilities (Table 3). Environmental organisations and charities have a vital role to play: promoting healthy engagement with nature as part of their missions. Employers also can act by focusing on work-life balance, and activities that contribute to well-being. Core intervention priorities should thus centre on hard-to-reach populations and cohorts, and those for whom current policies and treatment are struggling to find solutions. This includes those in certain age groups, such as children and the elderly, and those suffering from lifestyle-related conditions, such as obesity, type 2 diabetes, loneliness, and mental ill-health. These interventions are variously called Nature-Based Interventions (NBIs), Wise Psychological Interventions (WPIs), and Positive Psychological Interventions [17,143,144], and offer opportunities for individual as well as collective restoration [29,145].

The risk of death is reduced and longevity increased through social group membership and social support [146,147], through interesting and healthy diets [74,148], through cognitive engagement with craft and mindfulness/spiritual activities [149,150], through physically-active lifestyles, such as in the Sardinian mountains [24], Okinawa [151], Harvard alumni [41], and Caerphilly men [42]. Combinations of all can lead to exceptional longevity [152,153,154,155]. It thus appears that extreme longevity is promoted by the habitual consumption of healthy food, daily physical activity, an engagement with nature, strong social capital, and cognitive engagement. These also offer the prospect of living well and with contentment.

We propose twelve research questions to focus action on children, adults, healthy longevity, and redesign.

### 9.1. Children

1. Young children are becoming increasingly socially-disconnected, inactive, and eating badly: what green mind interventions would work best for 5–11 year olds?
2. How protective would such interventions be across the lifecourse?

### 9.2. Adults

3. How can immersive-attention activities be promoted to produce health and well-being improvements across whole populations (including the disadoption of unhealthy habits)?
4. What are the best policies for local and national governments to implement that would aid such adoption and disadoption?
5. What priority activities and behaviours should be promoted at stressful transition points in the lifecourse (moving schools, transition to university, becoming a parent, marriage and divorce, deaths of friends and relatives, retirement, transition to social care)?

### 9.3. Longevity

6. What green mind interventions offer the greatest efficacy for health outcomes in elderly populations and social care settings?
7. How can interventions based on outdoor activity, social interactions, good food, and cognitive engagement be best implemented?
8. What can be learned from cultures where healthy living continues into the 8th–11th decades of the lifecourse (such as in the longevity hotspots of Nagano and Okinawa in Japan)?

### 9.4. Redesign

9. What well-being and health outcomes could be delivered in hospitals and care settings if green and prosocial design was implemented?
10. How much real income could be saved by hospitals from promoting behaviours that prevented the need for treatment?
11. How can human settlements be redesigned to increase engagement with existing green spaces?
12. Could some solutions to climate change rest in the green mind and its capacity to drive low consumption behaviours?

We conclude by setting out a ten-point call to action based on compelling supportive scientific evidence about the influence of lifestyle and behaviour on short- and long-term well-being (Table 4). None of this will be easy to achieve at the population level. It will require behaviour change and habit formation; it will require changes in infrastructure, policies, and resourcing. But every small contemplative undertaking can lead to the gradual formation of a green mind. It just needs relentless persistence long enough to form the habit. As indicated above, it will be institutions and communities that are likely to reach the largest number of people quickly. There could be emphasis on nature, social, and craft engagements in neighbourhoods, schools, care homes, and health care facilities. Environmental organisations and charities could play a vital role in promoting healthy engagement with nature as part of their missions. Every child could be outdoors every day; every older person in a care home could sit in a garden. Every economy could be green and prosocial. Now is time for a new ethic: the economy is the environment. Nature will survive us all.

## 10. Conclusions

### Comments on the Green Mind

Nudge tactics and policy changes have now made it possible in some countries to imagine the reality of an entirely tobacco-free culture, or where some water and air systems are pollution-free. In others, hunger has been dramatically reduced. What level, thus, of ambition is possible when considering policies and practices that could mostly solve lifestyle-related conditions and diseases typical of modern, high material-consumption cultures, utilizing the Green Mind Theory? Is it possible to imagine substantial reductions in incidence of mental ill-health, obesity, type 2 diabetes, loneliness, physical inactivity, and cardiovascular disease through the adoption of resilient *green mind* habits, fostering prosocial and greener economies across whole populations? We suggest Green Mind Theory offers some new opportunities to address these pervasive challenges by suggesting new routes to raising the well-being baseline of whole populations though the adoption of healthier habits and behaviours.

## Figures and Tables

**Table 1:** The annual costs of the health externalities arising from modern lifestyles, U.K.

Condition	Proportion of Population Currently Affected	Number Currently Affected	Full Annual Cost to Economy (£ Billion)
Mental ill-health	18% of adults 10% of children	8.8 million	105.0
Dementias	13% of >65 year olds	0.75 million	20.0
Obesity	26% of adults 15% of children	13 million adults 1.9 million children	20.0
Physical inactivity	20% of adults completely inactive	10 million adults	8.2
Diabetes (type 2)	5% of adults	2.9 million	29.0
Loneliness	30% of >65 year olds	0.9 million	40.0
Cardiovascular disease (including hypertension and strokes)		1.84 million in-patient episodes: 180,000 deaths	22.6
Total (assuming all costs independent and additive)			244.8
Total costs (assuming one quarter of costs double-counted through co-morbidities)			183.6

Note: obesity costs are assumed to be the same for adults and children. The annual health costs of obesity alone in the U.S. are $147 billion [49]. Source: Pretty et al. [13].

**Table 2:** Ten differences between mind typical of modern affluent culture and the green mind.

Modern Mind in Affluent Consumer Culture	Green Mind
Too many daily red alerts (first arrows)	Balance of blue and red brain
Chatter of second arrows increases stress, suffering, and anxiety	Second arrows are suppressed and suffering reduced
Immersion-attentiveness forced out of daily life	Immersion-attentiveness deliberately increased by daily habits and routines
Over-alert status of SNS-HPAA causes secondary health problems	PNS activates blue brain to engage in self-healing
Tendency towards mental ill-health	Tendency towards health and well-being
Empathy forced out by over alert red brain	Green mind is more prosocial and empathetic
Inactive, sedentary lifestyles	Regularly active
Disconnected from nature and sensate green places	Regular user of green places through green exercise
Some conditions and diseases only appear treatable through medication (or not at all)	Dose of green mind approaches can be therapeutic
Tendency towards increased material consumption for consolation and interest	Tendency towards non-material consumption and sustainable material consumption for well-being and long life

Notes: (i) SNS-HPAA is the sympathetic nervous system and hypothalamus pituitary adrenal axis; PNS = parasympathetic nervous system; (ii) we use the term “arrows” to refer both to external stimuli (alerts) and internal responses (first arrows are received by the amygdala and are unavoidable; second arrows comprise how we feel in response to first arrows).

**Table 3:** Nature, social, and craft engagements that build the green mind.

**Nature Engagements**	Activities that deliver health benefits in nature include walking, gardening and allotmenteering, fishing, rock climbing, bike/horse riding, outdoor tai chi/yoga, beach holidays, outdoor swimming, surfing river bores, watching sunsets or waves, dog walking, pigeon-racing, pilgrimage walking, bird watching, park running, and fen skating.
**Social Engagements**	Socially-based activities low in material consumption yet delivering health benefits include drama and song/choral groups, dance groups (ballroom, Morris dancing, Highland dance), coffee mornings, carol singing, conservation volunteering, participative prayer, book groups, curating social media online, bell-ringing, dance/night-clubs, fairs and fetes, parades and carnivals, horticulture societies, community supported agriculture groups, pop-up music festivals, folklore ceremonies (mud racing, cheese rolling, Halloween, bonfire night, beating the bounds, horn dance, tar barrel rolling, apple day, and rush bearing).
**Craft Engagements**	Craft activities that deliver attention and immersion, bringing further well-being benefits, include painting, drawing, writing, calligraphy, baking, jam-making, carpentry, home repairs and improvements, knitting, needlework, quilting, crosswords, mindfulness and meditation, tai chi/yoga, jewellery making, boat-building, craft beer brewing, wine-making, pottery, stone masonry, dry-stone walling, and hedge-laying.

**Table 4:** Ten calls to action for the green mind.

Every child outdoors every day.
Every adult physically-active every day.
Every adult learning a new skill or craft throughout life.
Every care home with a garden.
Every hospital redesigned on greener, prosocial principles.
Every natural environment promoted for some human use.
Every person able to access green, social and talking therapies.
Every person engaged in neighbourhood groups for social interaction.
Every kilogramme of fossil fuel left in the ground.
Every economy green and prosocial.
